# Double blanket effect caused by two layers of black carbon aerosols escalates warming in the Brahmaputra River Valley

**DOI:** 10.1038/srep03670

**Published:** 2014-01-14

**Authors:** P. R. C. Rahul, R. L. Bhawar, D. C. Ayantika, A. S. Panicker, P. D. Safai, V. Tharaprabhakaran, B. Padmakumari, M. P. Raju

**Affiliations:** 1 Indian Institute of Tropical Meteorology, Pune, India; 2 Department of Atmospheric and Space Sciences, University of Pune, Pune, India

## Abstract

First ever 3-day aircraft observations of vertical profiles of Black Carbon (BC) were obtained during the Cloud Aerosol Interaction and Precipitation Enhancement Experiment (CAIPEEX) conducted on 30^th^ August, 4^th^ and 6^th^ September 2009 over Guwahati (26°11′N, 91°44′E), the largest metropolitan city in the Brahmaputra River Valley (BRV) region. The results revealed that apart from the surface/near surface loading of BC due to anthropogenic processes causing a heating of 2 K/day, the large-scale Walker and Hadley atmospheric circulations associated with the Indian summer monsoon help in the formation of a second layer of black carbon in the upper atmosphere, which generates an upper atmospheric heating of ~2 K/day. Lofting of BC aerosols by these large-scale circulating atmospheric cells to the upper atmosphere (4–6 Km) could also be the reason for extreme climate change scenarios that are being witnessed in the BRV region.

Black Carbon (BC) alters the global radiation budget by absorbing and scattering sunlight^1^, reducing the net solar radiative flux to the earth's surface; hence becoming a major contributor to current global warming. On a global scale the largest source of BC aerosols has been the South East Asia (SEA)^2^,^3^. The Brahmaputra River Valley (BRV) of Southeast Asia recently has been experiencing extreme regional climate change due to black carbon^4^. BC is distinctively different from the other forms of carbon and carbon compounds contained in atmospheric aerosols. BC has strong visible light absorption of an average 7.5 m^2^ g^−1^ at 550 nm^5^ with vaporization temperature near 4000 K^6^, aggregate morphology^7^ and is insoluble in water and common organic solvents^8^. The spherules that are formed in flames due to fossil fuel combustions aggregate by rapid coagulation^9^ and these spherules differentiate black carbon from planar graphite. The precipitation patterns especially in the South Asian countries have been witnessing changes^10^, due to the light absorption by BC leading to the warming of the atmosphere thereby increasing the atmospheric stability^11^,^12^. The BRV region has been experiencing extreme regional climate change with increasing surface temperature at a rate of 0.03°K/year from 1980–2005^4^ and increased precipitation at a rate of 4 mm/year^13^. Over India, there have been few observational studies, confined only to first 2–3 Km of the atmosphere providing fewer data profiles^14^,^15^,^16^, except one study with the balloon that reached to an altitude of 8 Km^16^. Unfortunately, there have been no aerial campaigns to map the vertical profiles of BC loading in BRV, prior to this study. Hence, this study is crucially important to understand the vertical stratification of the BC variability in the BRV region that has significant implications on the glacial melting in the Himalayan region.

## Results

**Figure 1:**
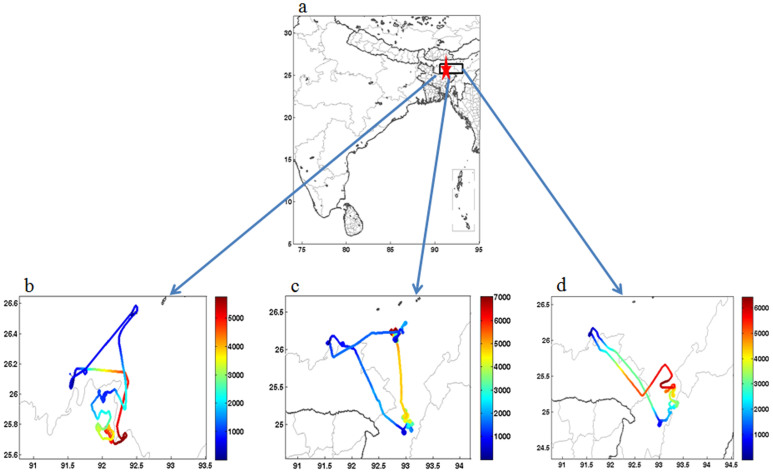
(a) The geographical map of India; where the star indicates Guwahati and the rectangular box indicates the area where the experiment has been conducted. Figure 1 (b), (c) and (d) show the flight tracks during 30^th^ August, 4^th^ September and 6^th^ September 2009. The color bar shows the altitude in meters. The figures were prepared using the MATLAB software.

**Figure 2:**
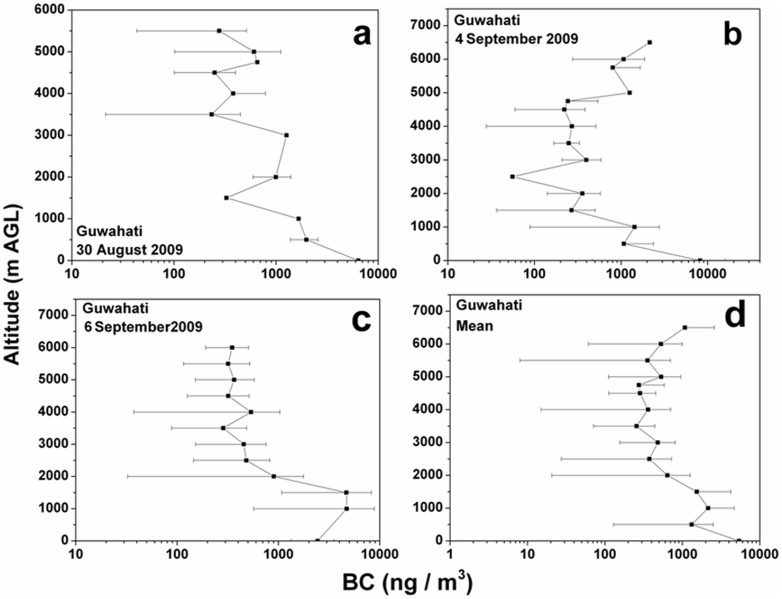
(a) Vertical profile of BC mass concentration with error bars over Guwahati during (a) 30^th^ August 2009 (b) 4^th^ September 2009 (c) 6^th^ September 2009 and (d) mean vertical profile.

**Figure 3:**
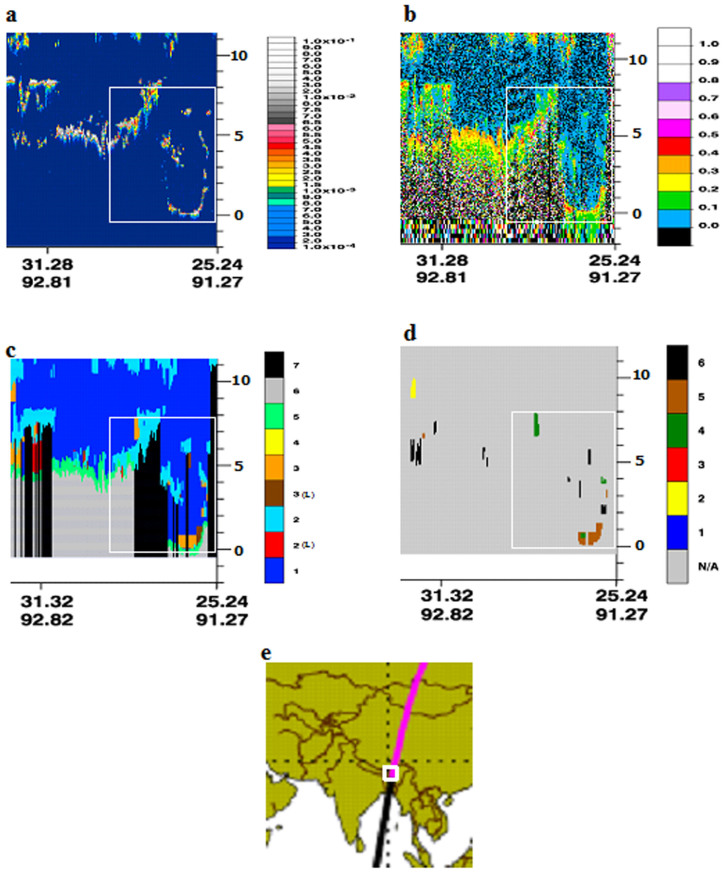
(a) CAPLISO (0–5 Km altitude range) profiles of 532 nm backscatter return signal during 9^th^ September 2009, respectively. The color scale in green, yellow and red represent low, medium and high loadings, respectively, while boundary layer clouds usually show up as gray or white. (b) DR profiles for the same date, smaller DR values indicating spherical particles like sulphates; black carbon and products of biomass combustion while higher DR values indicate non-spherical particles like dust. (c) VFM features during for the same date, Type: 0 = low/no confidence, 1 = clear air, 2 = cloud, 3 = aerosol, 4 = stratospheric feature, 5 = surface, 6 = subsurface, 7 = totally attenuated, L = low/no confidence. (d) Aerosol subtypes during the same transect, N/A = Not applicable, 1 = clean marine, 2 = polluted continental, 3 = clean continental, 4 = polluted dust and 6 = smoke. (e) Shows the CALIPSO transect over the area of study. The box in the figures highlights the CALIPSO transect over the region of study. CALIPSO profiles were obtained from (http://www-calipso.larc.nasa.gov/products/lidar/).

**Figure 4:**
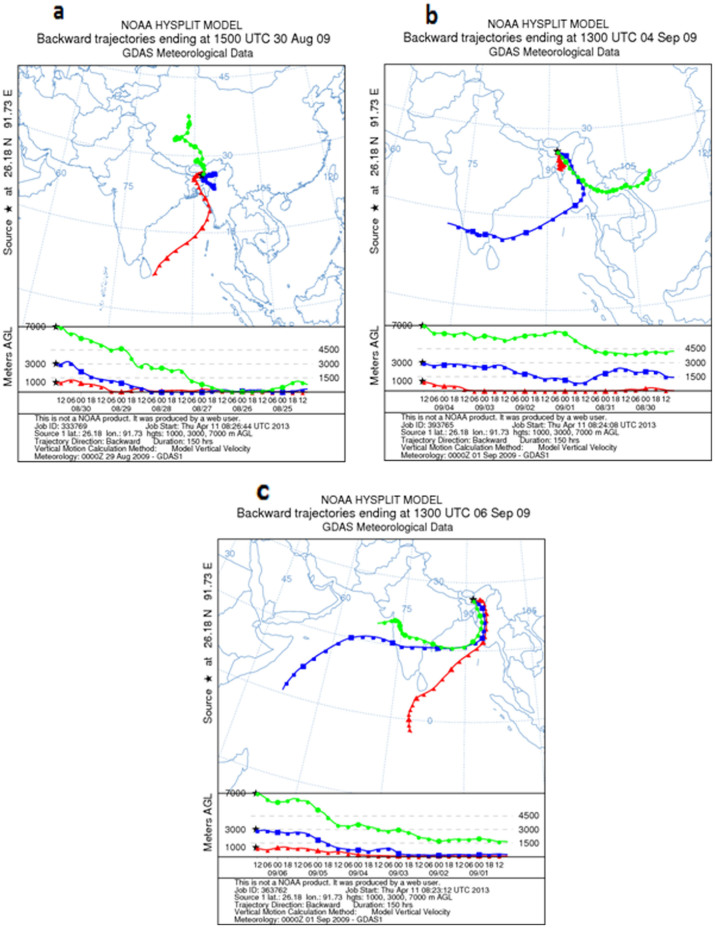
(a), (b) and (c) indicate 7-day NOAA HYSPILT back trajectories, these trajectories indicate the source regions along with the path traveled by air masses. The back trajectories were obtained from the NOAA Air Resources Laboratory (www.arl.noaa.gov/HYSPLIT.php.)

BC concentrations were measured during the aircraft maneuvers (Figure 1 b, c and d) over Guwahati (Figure 1 a), largest city in the BRV region. The observations were averaged for every 500 m height to obtain the mean value representing that altitude. Vertical profiles of Black Carbon Concentrations [BCC] with error bars during the 30^th^ August, 4^th^ and 6^th^ September 2009 are plotted in Figure 2 (a–c); while the mean profile is shown in Figure 2d. Figure 2a (30^th^ August 2009) shows the BCC of 6500 ng/m^3^ at 0 m (ground level) and then follows a decreasing trend till 1.5 Km with 350 ng/m^3^. From 1.5 Km there is relative increase till 3 Km with BCC at 1500 ng/m^3^, followed by increase and then decrease at 5.5 Km with BCC at 300 ng/m^3^. From Figure 2b (4^th^ September 2009) it is evident that the ground level BCC is 8500 ng/m^3^ and then continues to decrease till 1.5 Km at 300 ng/m^3^; with minimum BCC of 60 ng/m^3^ at 2.5 Km. From 2.5 Km to 6.5 Km there is steep increase of BCC values to 3000 ng/m^3^ at 6.5 Km. In Figure 2c (6^th^ September 2009), BCC of 2500 ng/m^3^ observed at the ground level with an increase to BCC at 7000 ng/m^3^ at ~1 Km followed by an decrease to 600 ng/m^3^ at 3.5 Km, and then BCC decreases to an average value of 250 ng/m^3^. The mean vertical profile of Figure 2 a, b and c is plotted in Figure 2d. The figure reflects a surprising pattern i.e., the BCC of 1000 ng/m^3^ at higher altitudes (5–6.5 Km) is 80% that of the observed BCC of 1200 ng/m^3^ at near surface (500 m) with ‘concave curve’ like variability, in-between. Since the annual average data show BC lifetimes is in the range of 4–12 days^17^, we have considered the Cloud-Aerosol Lidar and Infrared Pathfinder Satellite Observations (CALIPSO) transect that passed over Guwahati, though not exactly on the dates of the campaign (4^th^ and 6^th^ of September 2009) but on the 9^th^ September 2009. The CALIPSO profiles during 9^th^ September 2009 are shown in Figure 3(a–d); it is important to note that all the profiles have been obtained along the CALIPSO transect as shown in Figure 3e. The profiles considered are Total Attenuation Backscatter (only the 532 nm profiles are shown; the 1064 nm profiles are similar), Depolarization Ratio (DR), Vertical Feature Mask (VFM) and aerosol subtypes. Figure. 3(a) clearly shows the presence of aerosol plumes increasing in density latitude-wise between 3 and 5 Km and then appears as a patch at altitudes 6–7 Km. The DR profiles are shown in Figure 3b, the values are between 0.1–0.3, indicating spherical particles like sulphates; black carbon and products of biomass combustion. The VFM profiles along the track show the values between 2 and 3, indicating aerosols along the latitudinal variation as shown in Figure 3c; while Figure 3d shows the aerosol type phase profiles and the values of 4 & 5 indicate polluted dust and smoke aerosols were predominant along the transect. Hence, all the observed values of the CALIPSO profiles indicated the presence of black carbon in the atmosphere during the period of the aircraft measurements. Further, since the spatial distribution of BC aerosols in the atmosphere is significantly influenced by the prevailing winds^18^, the Hybrid Single-Particle Lagrangian Integrated Trajectory (HYSPLIT) back trajectories are shown in Figure 4 a–c. The back trajectories are over a period of 7 days prior to the date of aerial observations; the air parcel trajectories show the long-range transport from the North western region of the Asia Pacific and also from the Indian Ocean via the continental land mass.

## Discussion

**Figure 5:**
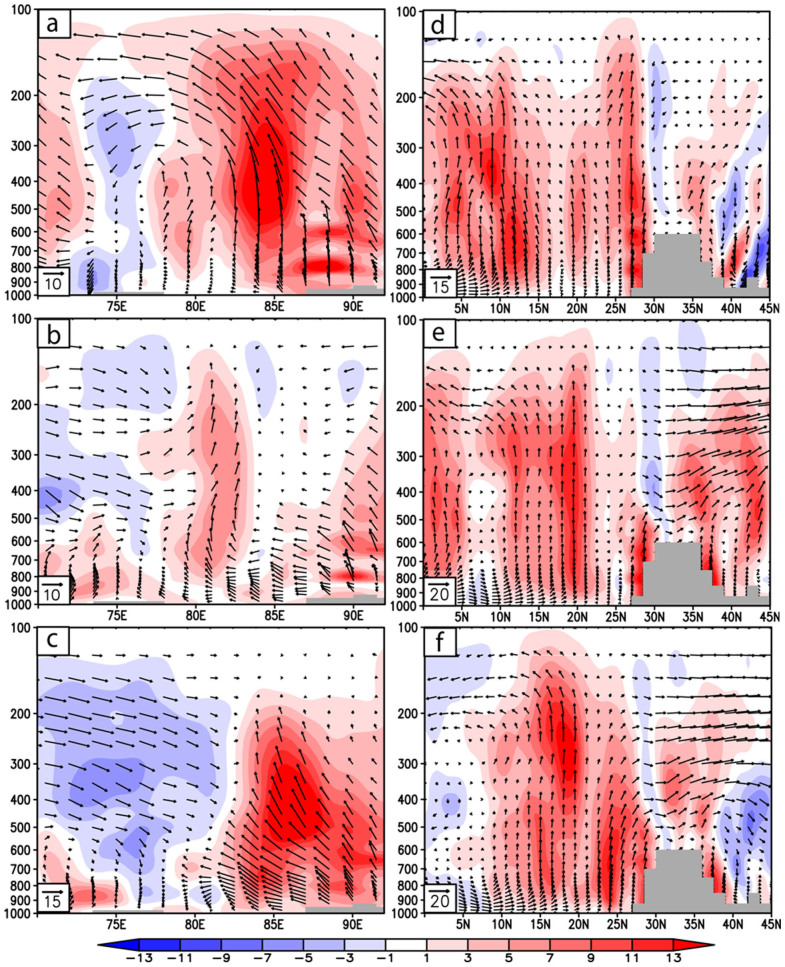
(a), (b), (c) represent the Walker circulation (zonal and vertical winds are averaged between 25N-27N) during 30 August, 4^th^ Sep and 6^th^ September 2009 respectively while (d), (e) and (f) represent the Hadley circulation (meridional and vertical velocity fields are averaged between 80E-92E). The vertical velocity (x -100 hPa/s) is shown by shading where red (blue) indicates upward (downward) motions. Gray portions indicate the Tibetan Plateau.

**Figure 6:**
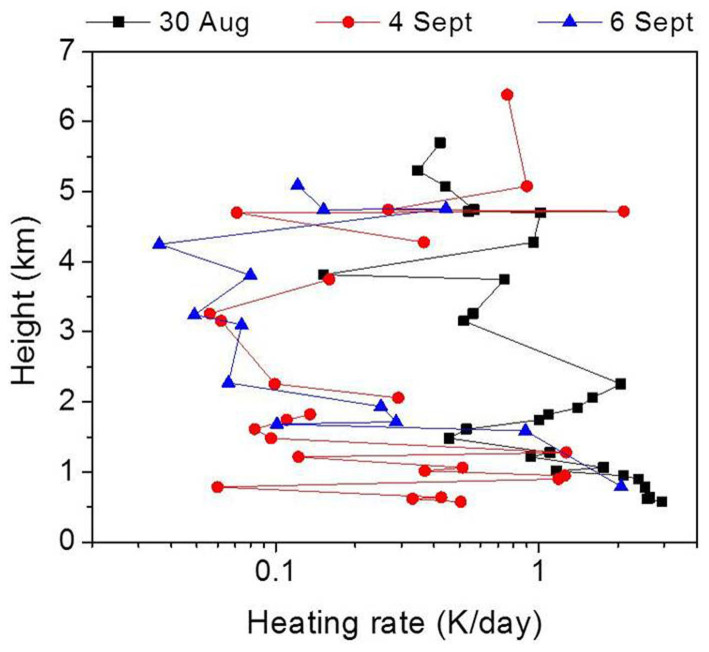
Vertical heating rates for 30^th^ August, 4^th^ and 6^th^ September 2009.

Globally, almost all the studies over China^19^, India^16^, Europe^20^ and Pacific^21^ conducted so far with aircrafts to study the BC variability along the altitudes more or less showed a similar pattern of variability, i.e., a progressive increase in the BC up to the boundary level and then subsequently decreased with increase in the altitude. It is often seen that the small dilution in the surface concentration due to increased convective mixing and deepening of the atmospheric boundary layer results in a near steady or decreasing mode of BC to an atmospheric height of 3 to 3.5 Km^22^. But what happens from 3 to 6.5 km in this specific experiment is interesting to observe; from the mean profile it becomes distinctly clear that from 3.5 km to 6.5 Km there is a rise in the BC concentrations. In order to investigate the cause for the increase in the BC at higher altitudes we consider the associated southwest monsoon Walker and Hadley circulation patterns (part of the large scale overturning circulation). Figure 5 (a – f) shows the vertical velocity (shading) and atmospheric circulation along the longitude pressure (x-p) section (monsoon Walker cell, Figure 5 (a–c)) and latitude pressure (y-p) section (monsoon Hadley cell, Figure 5(d–f)) respectively for the study period. For (x-p) sections the zonal and vertical winds are averaged for latitudes (25°N–27°N) and for the (y-p) sections the meridional and vertical velocities are averaged over northeast Indian region (80°E–92°E). Strong ascending motions characterize the overturning circulations over the northeast region extending from surface unto almost upper tropospheric levels with subsidence over western India and southern tropical Indian Ocean. But the strongest updrafts over the northeast sector are predominantly confined unto the mid-tropospheric (500 hPa) level for both latitudinal as well as longitudinal circulation cells. From figure 5 (a–c) it can be observed that over the eastern Himalayan foothills (~90°E), the upward motions show dual vertical maxima at around 2 Km (800 hPa) and 4–5 Km (600–500 hPa) with the intensity of vertical velocity decreasing progressively above mid-tropospheric levels. Along with the strong ascending motions, eastward bending of atmospheric flow is also noted at various tropospheric altitudes. Over the location of study (Guwahati, 26°11′N, 91°44′E) the intense upward motion at low to mid-levels is instrumental in transporting BC particles from surface to higher altitudes and the atmospheric motion above mid-troposphere is such that it helps in accumulation and thus trapping of BC particles at an altitude of 4–6 Km. The HYSPLIT 7-day back-trajectories (Figure 4) also shows that the parcels/particles from an altitude of 1.5 km or 3 km over the continental regions are transported to an altitude of 6.5 km over BRV region by the large-scale monsoon overturning circulations (Figure 5). Hence, ascending branches of the monsoon Walker and Hadley circulation help in creating a second layer of high BC concentrations, apart from the first layer at the surface caused by the anthropogenic, fossil fuel combustion and local emission processes. Thus, the BC concentrations at higher altitudes not only contain BC from long-range transport from over the oceanic regions (as we see in the HYSPILT back trajectories), but also would contain BC from the surface through the transport induced by the monsoon Walker and Hadley cells. From the Hadley circulation plots, it is evident that the BC aerosols are being lofted onto the Tibetan Plateau (30°–35°N) and well into China. This could be the process by which black carbon aerosols had been deposited and have been found on 5 locations on the Tibetan Plateau at altitudes of 4–6 Km, such depositions has been proved to substantially enhance glacial melting^23^. The vertical heating rates for the observed BC concentrations is estimated from the Santa Barbara discrete ordinate radiative transfer model (SBDART^24^) and are shown in Figure 6. The regression analysis showed a strong positive correlation of 0.78, 0.66 and 0.9 between BC heating and BC mass during 30^th^ August, 4^th^ and 6^th^ September 2009, respectively. 1 to 1 correlation is not expected since the heating rates also depend on the variability in temperature and pressure at each vertical level. Hence, from the SBDART model the rate of heating is directly proportional to the BC loading i.e., the heating in the lower atmosphere is ~2 K/day and again at altitudes of 5–6 Km it is ~2 K/day with a decline in between. Considering the high values of heating at the upper atmosphere, it becomes evident that the BC layer was above the clouds; since BC aerosol layers above the clouds tend to generate more heating then those below the clouds^25^,^26^,^27^. The results get corroborated when we consider the Figure 3b and 3c that show the DR values and VFM values. It is clear especially in the VFM profiles (with value 3) where the values indicate the presence of polluted dust (containing BC) at upper atmosphere, below which are clouds (with VFM values of 2).

The atmospheric radiative heating due to BC increases the atmospheric stability leading to a change in the precipitation patterns^28^,^29^,^30^ and when such heating occurs over glaciers/ice, it enhances the melting rate as proved in a recent study^31^. Several studies about the aerosol variability and the associated heating have been conducted over the Himalayan region^32^,^33^,^34^,^35^,^36^ and BC induced heating rates greater than 2 K/day have been reported over different Indian regions^16^,^37^. The results in the present work add to the understanding of the vertical BC loading and the subsequent heating at the higher altitudes. Such large-scale Indian summer monsoon mediated atmospheric circulation would enhance the accumulation of BC on the Tibetan Plateau thereby escalating the glacial melting.

Scenarios for future climate usually assume that most fossil fuels will be burned, causing additional global warming of at least several degrees Celsius^38^. In that event, most glaciers, worldwide, will be lost this century, with severe consequences for fresh water supplies^39^, as well as many other climate effects^40^.

Hence drastic cuts in the anthropogenic BC emission rates in the BRV region need to be reckoned with, before the BC accumulations in the upper atmosphere help fasten the melting/disappearance of the Tibetan glaciers^23^ due to the double blanket heating effects discussed in this paper. Future work would be to conduct more aerial campaigns and for longer duration in the BRV region to probe the vertical stratification of BC aerosols.

## Methods

As a part of the CAIPEEX program [for details^41^], a twin engine Piper Cheyenne N361 JC pressurized aircraft from Southern Ogallala Aquifer Rainfall (SOAR) Program, TX, USA was employed for observations during 30^th^ August, 4^th^ September and 6^th^ September 2009, over Guwhati (26°11′N, 91°44′E), which is located in the northeast Indian state of Assam, flight tracks are shown in Figure 1 b, c and d. Each flight lasted for about 2 to 3 h duration during the period 12:00 to 16:00 local time, as the boundary layer is generally fully evolved leading to the establishment of strong convective motions prior to the initiation of sampling. BC observations were carried out using an Aethalometer (Magee Sci. Inc., USA, AE-42) that was located in an unpressurized part of the aircraft. The sampling inlet of the Aethalometer was connected by using a 1.5 m long polyurethane tube (a non-conductive tubing to minimize the particle losses) to the common isokinetic Brechtel double diffuser inlet that was mounted on the pressurized part of the aircraft facing the airflow through a manifold within the fuselage. Standard procedures were adapted to calibrate the aircraft instruments during the campaign^42^. The measurement uncertainty in spite of the corrections used to the raw Aethalometer BC data was ± 15% or about 20 ng/m^3^ in terms of BC mass concentration. As such, the BC concentration at 6.5 km on 6^th^ Sep 2009 is an outlier and it was not considered (along with other such data points on the other two days) for mean BC profile at GHT. CALIPSO vertical profiles (TAB, DP, VFM and AST), are considered in this study (http://www-calipso.larc.nasa.gov/products/lidar/). CALIPSO flies in formation with the EOS Aqua and CloudSat satellites as part of the NASA Constellation^43^. CALIPSO provides global vertically resolved measurements of atmospheric aerosols (532 nm and 1064 nm) and its depolarization measurements at 532 nm provide high-resolution vertical profiles of aerosols^44^, CALIPSO profiles during 9^th^ September 2009 are presented. Black carbon (BC) induced Heating rates has been estimated over Guwahati on three experimental days viz. 30^th^August, 4^th^ and 6^th^ September 2009 during the instantaneous flight periods. We have used methodology^45^,^46^ explained in to estimate BC induced heating rates. The HYSPLIT (www.arl.noaa.gov/HYSPLIT.php) used are over a period of 7 days prior to the date of aerial observations. Surface BC concentrations were used in Optical properties of aerosols and clouds (OPAC) model^47^ to derive BC optical properties such as aerosol optical depth (AOD), single scattering albedo and Asymetry parameter at the surface. Subsequently, the surface BC aerosol parameters in conjunction with vertical BC profiles and temperature humidity profiles were used in Santa Barbara discrete ordinate radiative transfer model (SBDART)^24^ to derive Instantaneous vertical heating rates during observational period. The Walker and Hadley cells are obtained from the atmospheric winds from ECMWF interim reanalysis obtained from the ECMWF data server has been used to diagnose the large-circulation characteristics during the study period. The wind data is available at 1.5×1.5 horizontal resolution on 37 pressure levels.

## Author Contributions

P.R.C., designed the work, analyzed and wrote the paper, R.L.B. was also involved in the write up and making all the figures to the scientific reports standard, A.D.C. prepared the atmospheric circulations, A.P. prepared heating rates, T.P., P.K. provided the flight tracks while P.D.S. and R.M.P. provided the B.C values.
